# Enhanced Production of Androst-1,4-Diene-3,17-Dione by *Mycobacterium neoaurum* JC-12 Using Three-Stage Fermentation Strategy

**DOI:** 10.1371/journal.pone.0137658

**Published:** 2015-09-09

**Authors:** Minglong Shao, Xian Zhang, Zhiming Rao, Meijuan Xu, Taowei Yang, Hui Li, Zhenghong Xu

**Affiliations:** 1 The Key Laboratory of Industrial Biotechnology, Ministry of Education, Laboratory of Applied Microorganisms and Metabolic Engineering, School of Biotechnology, Jiangnan University, Wuxi, Jiangsu Province, 214122, P. R. China; 2 Laboratory of Pharmaceutical Engineering, School of Pharmaceutical Science, Jiangnan University, Wuxi, Jiangsu Province, 214122, P. R. China; CSIR, INDIA

## Abstract

To improve the androst-1,4-diene-3,17-dione (ADD) production from phytosterol by *Mycobacterium neoaurum* JC-12, fructose was firstly found favorable as the initial carbon source to increase the biomass and eliminate the lag phase of *M*. *neoaurum* JC-12 in the phytosterol transformation process. Based on this phenomenon, two-stage fermentation by using fructose as the initial carbon source and feeding glucose to maintain strain metabolism was designed. By applying this strategy, the fermentation duration was decreased from 168 h to 120 h with the ADD productivity increased from 0.071 g/(L·h) to 0.108 g/(L·h). Further, three-stage fermentation by adding phytosterol to improve ADD production at the end of the two-stage fermentation was carried out and the final ADD production reached 18.6 g/L, which is the highest reported ADD production using phytosterol as substrate. Thus, this strategy provides a possible way in enhancing the ADD production in pharmaceutical industry.

## Introduction

Steroid drug intermediates are widely used for the commercial production of pharmaceutical steroid drugs such as corticosteroids, mineralocorticoids, oral contraceptives, etc. Comparing with the chemical synthesis process, biotransformation of sterols (such as phytosterol, cholesterol and ergosterol) to steroid drug intermediates has its obvious advantage and has been widely used as a common and economical alternative method in the pharmaceutical industry [1, 2]. Since the discovery that microorganisms could degrade the side-chain of sterols to produce C_17_-ketosteroids [3], phytosterol has become a major raw material in pharmaceutical industry for its low cost with abundantly available and the ease of its transformation into steroid drug intermediates [1, 4–6]. Among these steroid intermediates, 4-androstene-3,17-dione (AD) and androst-1,4-diene-3,17-dione (ADD) are the two major products usually used as the starting material to prepare various kinds of important pharmaceutical steroids [7]. Many microbial strains have been reported capable of converting sterols to AD/ADD including *Aspergillus*, *Arthrobacter*, *Bacillus*, *Brevibacterium*, *Chryseobacterium*, *Fusarium*, *Gordonia*, *Nocardia*, *Pseudomonas*, *Rhodococcus* and *Streptomyces* [1, 8, 9]. Still, *Mycobacterium* remains one of the most efficient AD/ADD producers [1, 10].

The *Mycobacterium* bioavailability of phytosterol and the AD/ADD yield are limited due to the low sterol aqueous solubility [6]. Many efforts have been devoted to dealing with these shortcomings and improving AD/ADD production, e.g., the application of organic-aqueous biphasic systems, water-miscible organic co-solvents, cloud-point systems and liquid polymer based systems [11–14]. But, the activity and stability of the biocatalysts were inhibited by the toxic organic solvents, and consequently the application of these solvents on the sterols biotransformation was limited. Although the immobilization of mycobacterial cells onto silicone has been reported to improve the production of AD from sitosterol [15], the low sterol aqueous solubility inhibited the transformation efficiency, which limited the application of the immobilized cells. Since cyclodextrins (CDs) were firstly used to improve the sterols solubility in aqueous transformation, CDs have been extensively used in sterols transformation process for its obvious superiority [16, 17]. However, no report on the fed-batch of phytosterol and CDs for the transformation by *Mycobacterium* has been documented. Another major problem for *Mycobacterium* transformation of phytosterol is the long transformation duration and the low productivity due to the poor growth and a long lag phase time of *Mycobacterium* strain. Some scholars have studied the effect of media composition on the biotransformation of sterols to AD and ADD [18–20]. Despite all these studies, the long transformation period and the low productivity still needed to be solved.

Previously, *Mycobacterium neoaurum* JC-12 capable of transforming phytosterol to ADD as the main product was isolated from soil. In this study, in order to eliminate the lag phase of *M*. *neoaurum* JC-12, fructose used as the initial carbon source was selected and fermentation strategies with the fed-batch of phytosterol/CDs inclusion complex were applied to enhance the ADD production. Finally, the ADD production reached 18.6 g/L, which is the highest reported ADD production using phytosterol as substrate. This work is hoped pave the way to improve valuable ADD production from sterols.

## Materials and Methods

### Materials

ADD with the purity ≥99% was purchased from Sigma (USA). Substrate phytosterol used was obtained from Zhejiang DAVI biochemistry CO., Ltd (Zhejiang, China), which is composed of 47.5% β-sitosterol, 26.4% stigmasterol, 22.5% campesterol and 3.6% brassicasterol. Carboxymethyl-β-cyclodextrin (CM-β-CD), Methyl-β-cyclodextrin (Me-β-CD) and Hydroxypropyl-β-cyclodextrin (HP-β-CD) were obtained from Zhiyuan Biotechnology Co., Ltd (Shandong, China). All other chemicals were purchased from commercial sources.

### Microorganism and media

*M*. *neoaurum* JC-12 isolated from soil was maintained at 4°C on slant which contained the following (g/L): glucose 10, tryptone 10, beef extract 6, NaCl 10 and agar 20 (pH 7.0). The strain *M*. *neoaurum* JC-12 was grown at 30°C and 160 rpm on a rotary shaker in flasks (250 mL) with 50 mL seed medium with the following composition (g/L): glucose 10, tryptone 10, beef extract 6, K_2_HPO_4_ 3, MgSO_4_·7H_2_O 0.5 and MnCl_2_·4H_2_O5×10^−4^ (pH 7.0).

### Transformation of phytosterol

After grown in the seed medium for 48 h, the strain was inoculated with 10% volume into fresh transformation medium (100 mL in 500 mL flasks) with the composition as follows (g/L): glucose 20, tryptone 10, beef extract 6, K_2_HPO_4_ 3, MgSO_4_·7H_2_O 0.5 and MnCl_2_·4H_2_O 5×10^−4^ (pH 7.0). Then, the phytosterol or phytosterol/CDs inclusion complex was added into the medium to conduct the transformation reaction on a rotary shaker at 30°C and 160 rpm. Samples were withdrawn from culture broth at regular intervals and extracted by ethyl acetate.

### Effects of molar ratio (CDs:phytosterol) on ADD production

Six kinds of different CDs (α-CD, β-CD, γ-CD, CM-β-CD, Me-β-CD and HP-β-CD) on the effect of ADD production were carried out with the molar ratio of 1:1. Then, the different molar ratio of CDs to phytosterol on the phytosterol conversion efficiency and ADD production was further investigated.

### Effect of different substrate concentrations on ADD production

Six kinds of different concentrations of phytosterol (5 g/L, 10 g/L, 15 g/L, 20 g/L, 25 g/L and 30 g/L) in phytosterol/CDs inclusion complex were added into the transformation medium and the cultivation method is the same as above. As the phytosterol affects the OD_*600*_ measurement, the biomass was determined as the number of colony-forming units (CFU) per ml of cultural liquid [21].

### Effect of different carbon sources on cell growth

Different carbon sources (sucrose, maltose, lactose, fructose, glycerol and molasses) medium were prepared by replacing glucose in the transformation medium with the carbon sources at a uniform carbon mass concentration. As there was no addition of phytosterol, the biomass of the strains in different medium was determined by analyzing the OD_*600*_ of the cultural liquid.

### Two-stage fermentation and three-stage fermentation in 5-L fermentor

In a 5-L fermentor (Shanghai Baoxing Bio-Engineering Equipment Co., Ltd., China), we carried out the two-stage fermentation as follows. In the first stage, the *M*. *neoaurum* JC-12 was inoculated and cultivated in the fermentation medium with fructose as the carbon source but without phytosterol/CDs inclusion complex. At 24 h, which in accordance with the beginning of the second stage, phytosterol/CDs inclusion complex was added and glucose was fed as the carbon source at a proper time. After another 96 h, cultivation was ended. In the three-stage fermentation, the first and second stages were in accordance with the two-stage fermentation. At 120 h, which in accordance with the beginning of the third stage, phytosterol/CDs inclusion complex was added and the cultivation was ended until phytosterol concentration remained almost unchanged. The three-stage fermentation was also carried out in 5-L fermentor. The pH and DO were also controlled during the transformation process of the two and three-stage fermentation.

### Analytical methods

1 mL of sample was withdrawn from culture broth and extracted with 4 mL of ethyl acetate. After centrifugation, 2 mL of the supernatant was analyzed on a Shimadzu HPLC instrument equipped with a C18 column (Diamonsil C18, 5 μm particles, 250 mm×4.6 mm) and a UV/visible detector. ADD and AD were detected at 254 nm and the mobile phase composed of methanol and water (70/30, v/v). The flow rate was 1 mL/min and the column temperature was 30°C. Phytosterol was measured according to the Lieberman-Burchard color reaction [22]. The concentrations of glucose and fructose were also determined by HPLC using refractive index detector and Aminex HPX-87P column at 85°C with H_2_O as the mobile phase with a flow rate of 1 mL/min.

## Results and Discussion

### Selection and optimization of CDs to improve ADD production

Due to the hydrophobic cavity at the center and hydrophilic outer surfaces, CDs have been widely used to improve the substrate aqueous solubility in sterols biotransformation [17, 23, 24]. Among the six kinds of CDs investigated (α-CD, β-CD, γ-CD, CM-β-CD, Me-β-CD and HP-β-CD), Me-β-CD was found to be the most effective in enhancing the phytosterol conversion efficiency and improving the production of ADD in *M*. *neoaurum* JC-12 biotransformation (S1 Fig). Therefore, the different concentrations of Me-β-CD on ADD production were further studied.

**Fig 1:**
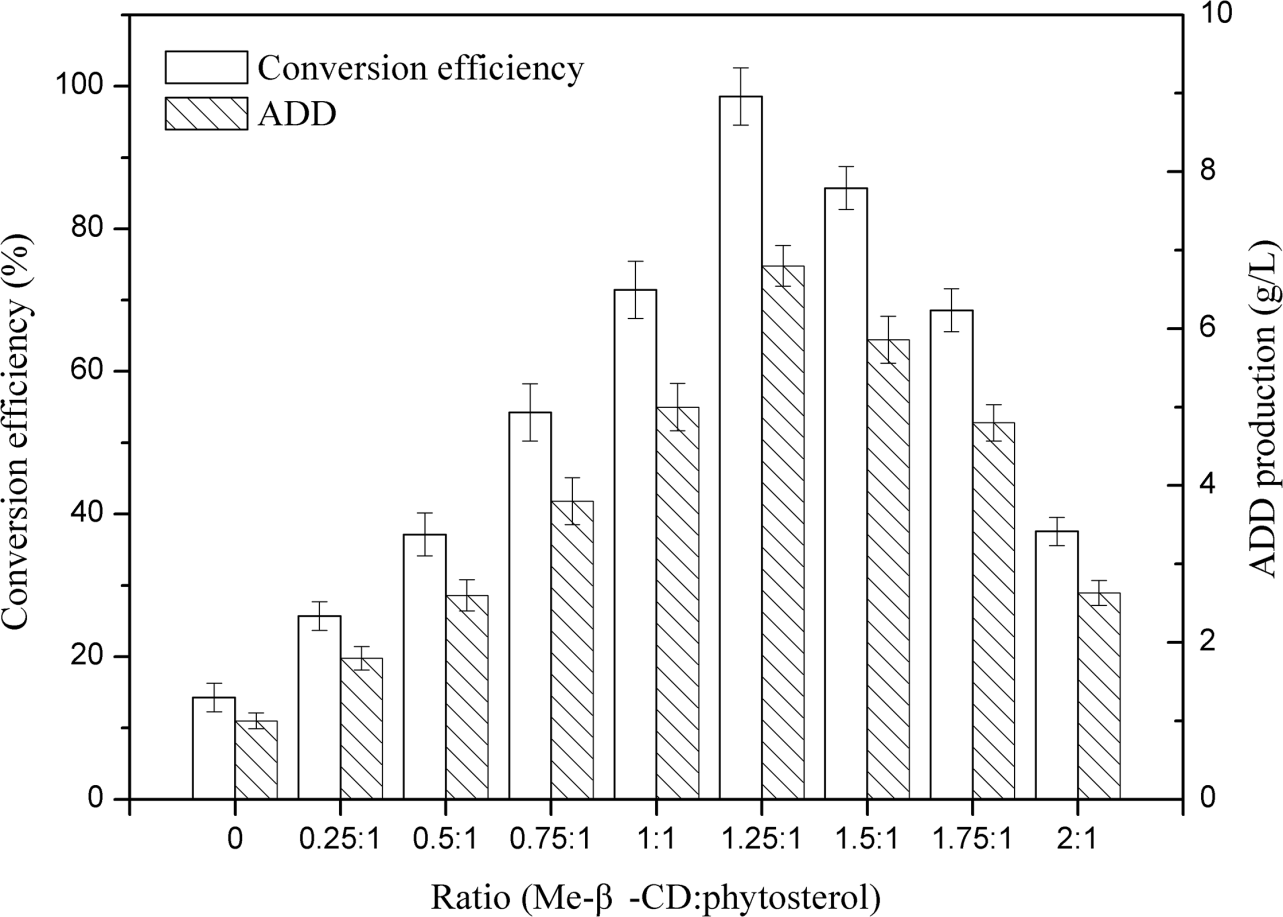
Effect of the molar ratio of Me-β-CD to substrate on phytosterol conversion efficiency and ADD production. All assays were performed in triplicate, standard deviations of the biological replicates were represented by error bars.

As shown in Fig 1, phytosterol conversion efficiency and ADD production increased accompanied by the increase of Me-β-CD concentrations in some extent. However, the conversion efficiency and ADD production decreased gradually when the Me-β-CD to phytosterol ratio was more than 1.25:1. The results indicated that high Me-β-CD concentration was toxic to *M*. *neoaurum* JC-12 and limited the transformation of phytosterol to ADD. Besides, at a high Me-β-CD concentration, phytosterol molecular was enclosed by Me-β-CD and “less accessible” for bioconversion, which in return inhibited the conversion efficiency [25]. The phytosterol conversion efficiency and ADD production were 97.15% and 6.56 g/L under the optimal molar ratio of 1.25:1. While, in contrast, the results of control samples without CDs were only 17.50% and 1.01 g/L, respectively. Therefore, the optimal molar ratio of 1.25:1 was used in the next study.

### Effect of phytosterol concentration on ADD production

**Fig 2:**
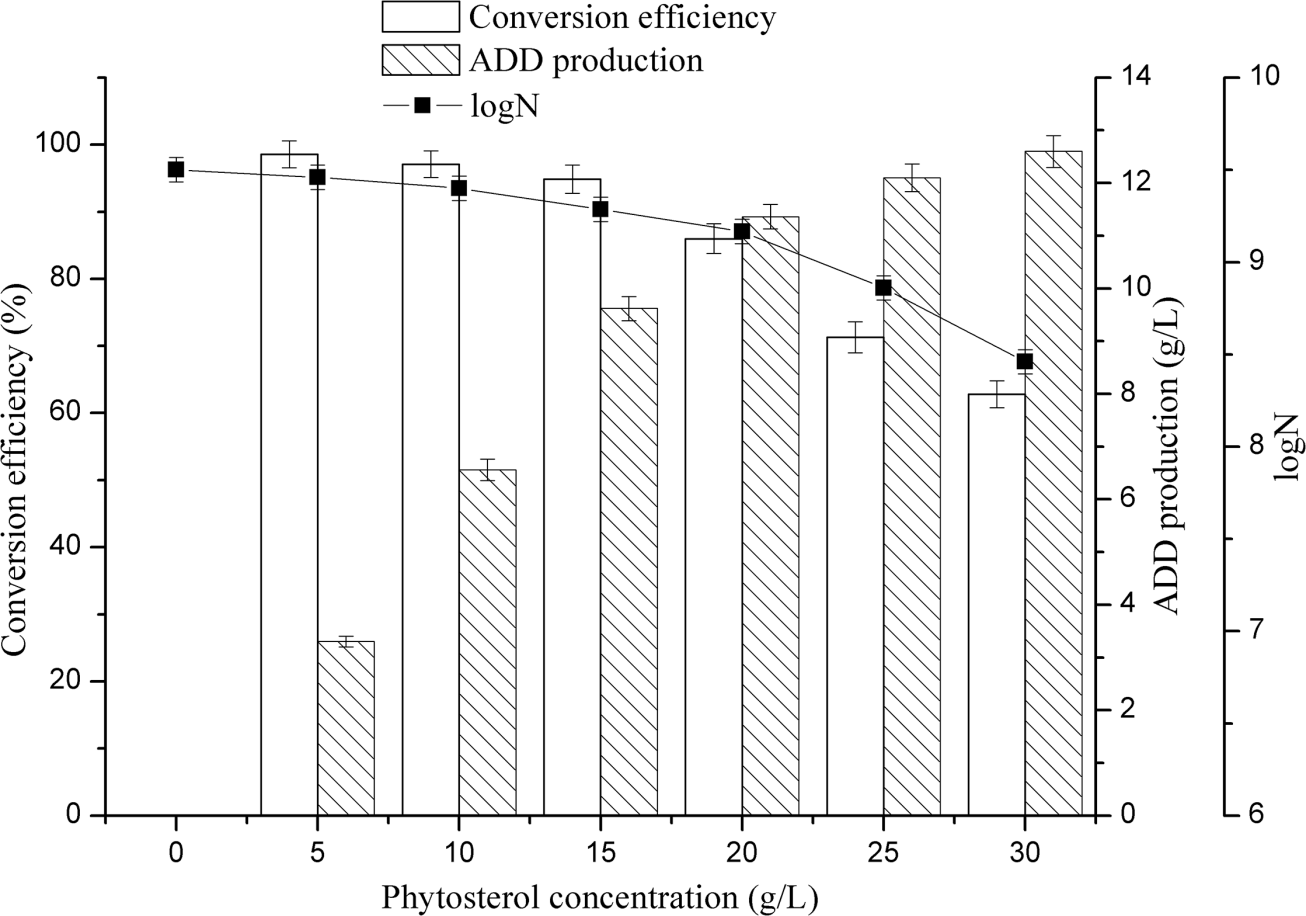
Effect of different substrate concentration on phytosterol conversion efficiency and ADD production. N, the number of CFU (colony forming units) per one milliliter of culture fluid. All assays were performed in triplicate, standard deviations of the biological replicates were represented by error bars.

The different initial phytosterol concentrations from 5 to 30 g/L on the conversion efficiency and ADD production were investigated in this study. As shown in Fig 2, at a lower concentration of phytosterol in the medium, higher phytosterol conversion efficiency and biomass was observed. While, with the increasing phytosterol concentration, the phytosterol conversion efficiency and the biomass decreased dramatically. This result was in accordance with the discovery by Gulla et al. (2010). They reported that the effect of different concentrations of soysterols on the bioconversion by *M*. *fortuitum* subsp. *fortuitum* NCIM 5239 indicated a steady decrease in the conversion to AD with increasing concentration of soysterols [26]. Besides, Roy et al. (1991) also reported the similar adverse effect at high concentration of the substrate [27]. Although the ADD production increased slightly with the increasing of phytosterol concentration, the phytosterol conversion efficiency decreased obviously which lead to the waste of the substrate and not suitable for industrial production. Therefore, overall considering the ADD production and the substrate conversion efficiency, 20 g/L phytosterol was selected as the optimum initial substrate concentration for the next study with 85.6% conversion efficiency and 11.3 g/L ADD production. The cell growth was inhibited under the high substrate concentration, which is generally caused by the inhibition of substrate on respiration chain [23].

### Effect of different carbon sources on cell growth

In order to avoid the substrate inhibition on cell growth and obtain high cell density, substrate was usually added into the fermentation medium after the cells grown for a certain time. In the biotransformation of dehydroepiandrosterone (DHEA) by *Colletotrichum lini* ST-1, the fine powder of DHEA was added into the medium after cultivating for 24 h [25]. The biotransformation of soybean phytosterols by *M*. *neoaurum* NwIB-01 was conducted with the addition of 15 g/L soybean phytosterols after strains were grown for 24 h in the fermentation medium in a 3.7-L bioreactor [28]. Therefore, in the first stage of the fermentation, high biomass was necessary for improving the productivity.

**Fig 3:**
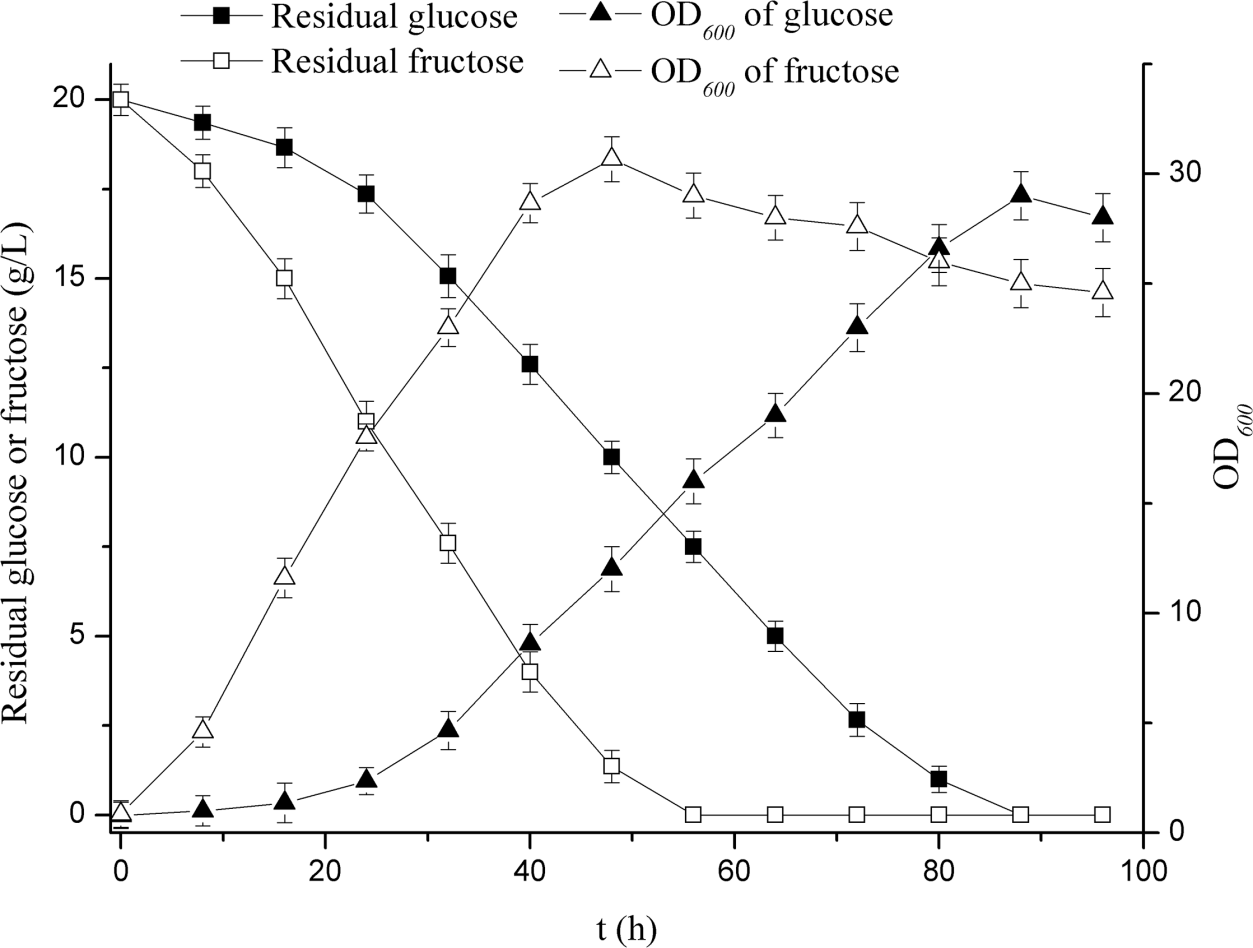
Effect of fructose and glucose on the cell growth of *M*. *neoaurum* JC-12. All assays were performed in triplicate, standard deviations of the biological replicates were represented by error bars.

In order to obtain high biomass of *M*. *neoaurum* JC-12 in the first stage of the fermentation, the effect of seven kinds of different carbon sources on biomass was investigated in this work. As shown in Table 1, among the carbon sources studied, fructose showed the obvious effect in improving the biomass and the OD_*600*_ value of fructose was 2-fold higher than that of glucose. As sucrose, maltose and lactose are disaccharide. These disaccharides have to be degraded into monosaccharide before they can be used for cell growth. Therefore, glucose and fructose supported a lot better cell growth than these disaccharides. Although the main component of molasses is sucrose, the molasses contains many other nutritional ingredients, such as amino acids, organic acids, inorganic compounds and vitamins [29], which are helpful for cell growth. So the molasses condition showed better cell growth than sucrose. The utilization of glycerol by *M*. *neoaurum* JC-12 was slowly, so the cell growth was at a low level. Compared the metabolic pathway of glucose and fructose in *M*. *neoaurum* JC-12, fructose is catalyzed by hexokinase into fructose-6-phosphate, while glucose is catalyzed by hexokinase into glucose-6-phosphate, then glucose-6-phosphate is catalyzed by phosphoglucose isomerase into fructose-6-phosphate. This is possibly why *M*. *neoaurum* JC-12 preferred utilize fructose than glucose, and as a result, fructose supported a lot better cell growth than glucose. Therefore, fructose was supposed to be an alternative of glucose in the first stage to obtain high biomass. The effect of glucose and fructose on the biomass was further studied in a 5-L fermentor. As seen in Fig 3, the biomass increased obviously in the first 48 h with fructose used as the carbon source when compared with glucose. Besides, fructose could eliminate the lag phase of *M*. *neoaurum* JC-12, which was supposed to decrease the long transformation period and enhance the low ADD productivity. Therefore, the effect of fructose on phytosterol transformation and ADD production was further investigated.

**Table 1:** The effect of different carbon sources on biomass of *M*. *neoaurum* JC-12.

	OD_*600*_						
t (h)	glucose	sucrose	maltose	lactose	fructose	glycerol	molasses
24	2.86±0.26	1.12±0.11	1.68±0.18	0.96±0.11	6.02±0.55	1.23±0.12	2.35±0.23
48	5.89±0.45	1.59±0.13	2.41±0.22	1.37±0.17	13.56±0.62	2.21±0.23	4.56±0.38

All assays were performed in triplicate, standard deviations of the biological replicates were shown.

### Two-stage fermentation

**Fig 4:**
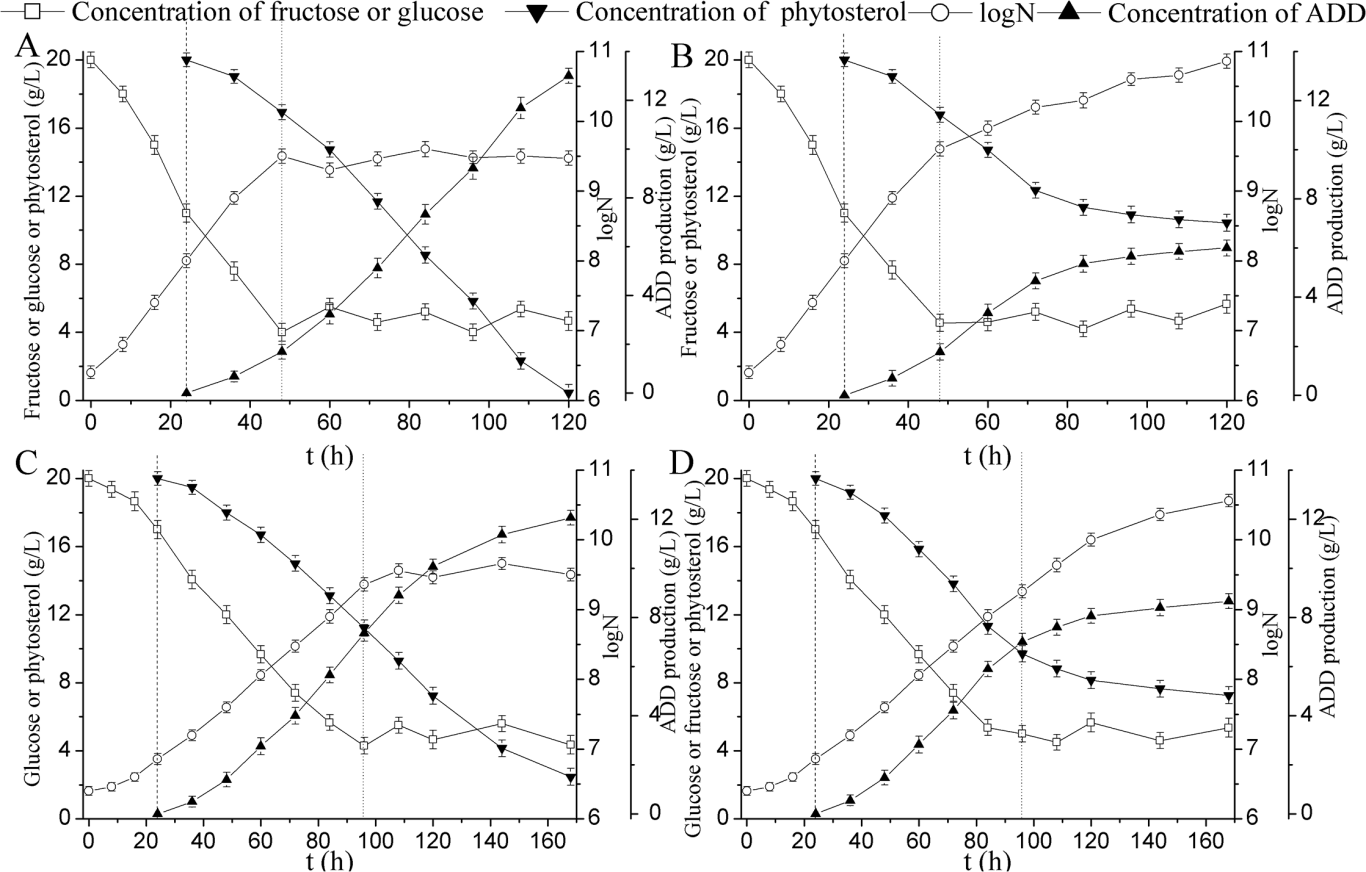
Two-stage fermentation in 5-L fermentor. Phytosterol/CDs inclusion complex was added at 24 h. (A) Fructose was used as the initial carbon source with glucose fed at 48 h. (B) Fructose was used as the initial carbon source with fructose fed at 48 h. (C) Glucose was used as the initial carbon source with glucose fed at 96 h. (D) Glucose was used as the initial carbon source with fructose fed at 96 h. N, the number of CFU (colony forming units) per one milliliter of culture fluid. All assays were performed in triplicate, standard deviations of the biological replicates were represented by error bars.

Based on the process of phytosterol transformation, two-stage fermentation was carried out to enhance ADD productivity in a 5-L fermentor. In the first stage, 20 g/L fructose was used as the carbon source to increase the biomass and eliminate the lag phase of *M*. *neoaurum* JC-12. At the beginning of the second stage (24 h), 20 g/L of phytosterol in phytosterol/CDs inclusion complex was added into the fermentation medium to carry out the bioconversion and the fructose or glucose was fed to maintain strain metabolism at a proper time. The stock solution concentrations of glucose and fructose were 200 g/L. In order to maintain the concentrations of glucose and fructose at about 5 g/L, the feeding rates of glucose and fructose were 0.2 g/(L·h) and 0.4 g/(L·h), respectively. As shown in Fig 4A, in the first 48 h, the biomass increased obviously and the lag phase was eliminated when fructose was used as the initial carbon source. With the glucose fed as the carbon source, the biomass maintained about the same, while the ADD production increased dramatically and reached 13.0 g/L at 120 h. It was supposed to increase the biomass during the fermentation by feeding fructose as the carbon source in order to enhance the ADD production. However, as seen in Fig 4B, although the biomass increased obviously during the fermentation, the ADD production remained at a low level compared with Fig 4A. All these results indicated that glucose fed as the carbon source was better than fructose in enhancing ADD production. This is mainly due to the cell aggregation level in fructose is significantly higher than in glucose, which possibly inhibited the cell respiratory function and the uptake of the substrate [30]. Different carbon sources may induce different metabolic activities or lead to different cell envelope characteristics, interfering with substrate mass transfer [4]. Further studies are needed to investigate the substrate mass transfer properties under different carbon sources. As shown in Fig 4C, although the ADD production could reach 12.0 g/L at 168 h when glucose was used as the initial carbon source and fed during the fermentation, the time needed for this process was longer compared with that in Fig 4A. The ADD productivity of Fig 4A was 0.108 g/(L·h), which was higher than that of Fig 4C (0.071 g/(L·h)). This is mainly due to the lag phase of *M*. *neoaurum* JC-12 was eliminated when fructose was used as the initial carbon source. When glucose used as the initial carbon source and fed with fructose, The ADD production remained at a low level (Fig 4D). In this sense, all of these results can be concluded as follows: (1) Fructose used as the initial carbon source can decrease the fermentation period by eliminating the lag phase and enhance the ADD productivity. (2) Glucose fed as the carbon source is better than fructose in improving ADD production.

### Three-stage fermentation

**Fig 5:**
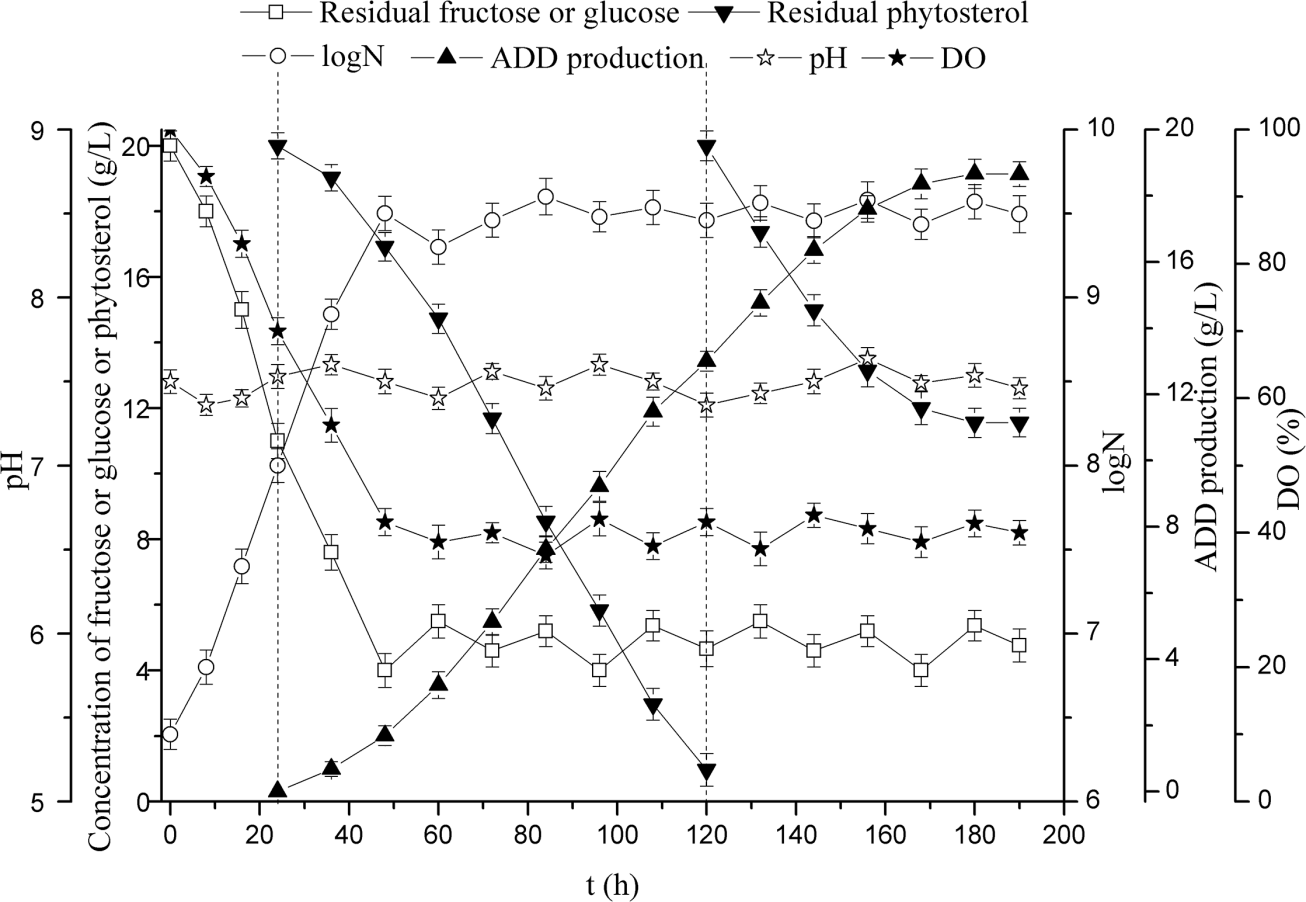
Three-stage fermentation in 5-L fermentor. Phytosterol/CDs inclusion complex was added at 24 h and 120 h, respectively. Glucose was fed at 48 h. N, the number of CFU (colony forming units) per one milliliter of culture fluid. All assays were performed in triplicate, standard deviations of the biological replicates were represented by error bars.

As shown in Fig 4A, at the end of the two-stage fermentation, the phytosterol transformation capacity still remained at a high level. Therefore, in order to further improve ADD production, three-stage fermentation was carried out on the basis of two-stage fermentation. In the three-stage fermentation, the first and second stages were the same as the two-stage fermentation. At the beginning of the third stage, 20 g/L of phytosterol in phytosterol/CDs inclusion complex was added when phytosterol was almost degraded. It can be seen in Fig 5, with the addition of 20 g/L phytosterol at the beginning of the third stage, the ADD production increased in some extent and reached 18.6 g/L. Compared with the two-stage fermentation, although the ADD productivity of the three-stage fermentation decreased to some extent (0.098 g/(L·h)), the ADD production increased from 13.0 g/L to 18.6 g/L (Table 2). It has been reported that lecithin could enhance the biotransformation of cholesterol to ADD, and the final ADD yield was 59% (w/w) [31]. Shen et al. (2012) showed that HP-β-CD appreciably increased the ratio of ADD to AD, the reaction rate and the molar conversion. In the presence of HP-β-CD, with 0.5 g/L phytosterol as the substrate, the ADD proportion of three different strains increased by 38.4%, 61.5% and 5.9% compared with the control experiment [32]. To improve the ADD production, a resting cell biotransformation of phytosterol to ADD in cloud point system was carried out, and the ADD production up to 12 g/L was achieved using 25 g/L phytosterol [33]. The 3-ketosteroid-Δ^1^-dehydrogenase was augmented in *M*. *neoaurum* NwIB-01 to increase the production of ADD, and the final ADD yield reached 4.23 g/L with 15 g/L phytosterol used as the substrate [28]. Saab et al. (2013) showed that immobilization of *Mycobacterium* species on the dried fruit of *Luffa cylindrica* increased the ADD production up to 0.30 g/L [34]. Besides, the recombinant *Bacillus subtilis* harboring the 3-ketosteroid-Δ^1^-dehydrogenase was used to transform AD to ADD in our previous study, and the ADD yield reached 0.66 g/L [35]. Despite all this study, to our knowledge, this is the highest reported ADD production using phytosterol as substrate.

**Table 2:** Comparing of microbial production of ADD using different fermentative strains or biocatalysts.

Strains and biotransformation mode	Substrates	Biotransformation duration (h)	Final concentration (g/L)	Productivity (g/(L·h))	References
**Fermentation**					
*Mycobacterium* sp. NRRL-B3683	Soybean phytosterols	120	0.70	0.006	[6]
*Arthrobacter simplex* and *Mycobacterium* sp. NRRL-B3683	Cholesterol	336	12.8	0.038	[36]
*Chryseobacterium gleum*	Cholesterol	24	0.08	0.003	[8]
*Mycobacterium* sp. DSMZ2967	Wood sterols	96	0.70	0.007	[18]
*Nocardia* sp.	Cholesterol	96	0.11	0.001	[19]
*M*. *neoaurum* NwIB-04	Soybean phytosterols	96	4.94	0.051	[28]
*Mycobacterium* sp.	Phytosterol	96	0.71	0.007	[37]
*Gordonia neofelifaecis*	Cholesterol	96	0.44	0.005	[38]
*M*. *neoaurum* NwIB-01MS	Phytosterol	144	5.57	0.039	[39]
*M*. *neoaurum* JC-12	Phytosterol	190	18.6	0.098	This study
**Biocatalyst**					
*Mycobacterium* sp. NRRL-B3683	Phytosterol	144	12.0	0.083	[33]
*Mycobacterium* sp. DSM 2966	Phytosterol	96	0.30	0.003	[34]
*B*. *subtilis* 168	AD	10	0.66	0.066	[35]
*B*. *subtilis* WB600	AD	48	0.45	0.009	[40]

Many efforts have been made in improving the substrate solubility and enhancing AD/ADD production, but the report of the medium composition on enhancing the ADD production was limited. The effect of different nitrogen sources on the bioconversion of sterols with *Mycobacterium* sp. has been investigated, but there are no special difference on the yield and biomass activity was observed [18]. It was reported that the ADD production by *Nocardia* sp. was hampered in the presence of glucose, which interfering with substrate mass transfer [19]. However, in this study, it was found that feeding glucose as carbon source could enhance the ADD production by *M*. *neoaurum*, which was different from the effect in *Nocardia* sp. [19]. Besides, in this study, we found that the ADD production was hampered in the presence of fructose, which was in accordance with the effect of glucose in *Nocardia* sp. [19]. These results mainly due to the different carbon sources induce different metabolic activities or lead to different cell envelope characteristics [4, 30]. This is the first report of applying three-stage fermentation to improve the ADD production by *M*. *neoaurum*, and further researches are required to investigate the different metabolic mechanisms and the substrate mass transfer properties of *M*. *neoaurum* under different carbon sources.

## Conclusion

In this study, fructose used as the initial carbon source was found capable of increasing the biomass and eliminating the lag phase of *M*. *neoaurum* JC-12 when compared with glucose. In the two-stage fermentation, the ADD productivity increased from 0.071 g/(L·h) to 0.108 g/(L·h) as the fermentation period was decreased from 168 h to 120 h. In the three-stage fermentation, 20 g/L phytosterol was added to further improve the ADD production at the end of the second stage and the final ADD production reached 18.6 g/L, which is the highest reported ADD production using phytosterol as substrate.

## Supporting Information

S1 Fig Effect of different CDs (α-CD, β-CD, γ-CD, CM-β-CD, Me-β-CD and HP-β-CD) on ADD production. CK, blank control without CDs. All assays were performed in triplicate, standard deviations of the biological replicates were represented by error bars.
(DOC)
