# Supplementary material for: Enhanced Production of Androst-1,4-Diene-3,17-Dione by Mycobacterium neoaurum JC-12 Using Three-Stage Fermentation Strategy
Source: PLoS One. 2015 Sep 9;10(9):e0137658. doi: 10.1371/journal.pone.0137658 (PMC4564235; doi:10.1371/journal.pone.0137658)
Supplement: S1 Fig — CK, blank control without CDs. All assays were performed in triplicate, standard deviations of the biological replicates were represented by error bars. (DOC) [file pone.0137658.s001.doc]

**S1 Fig. Effect of different CDs (α-CD, β-CD, γ-CD, CM-β-CD, Me-β-CD and HP-β-CD) on ADD production.** CK, blank control without CDs. All assays were performed in triplicate, standard deviations of the biological replicates were represented by error bars.

**
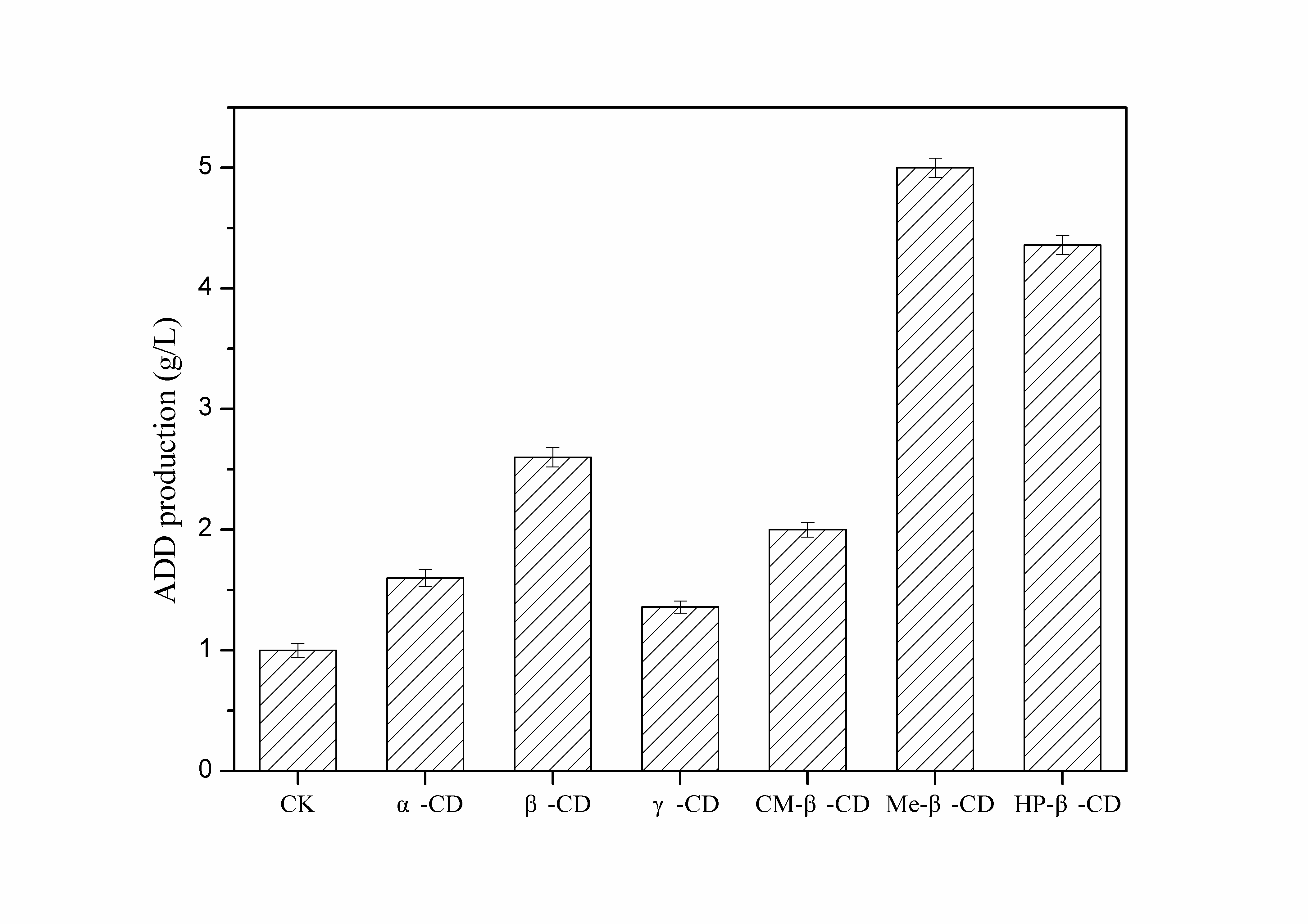
**
